# First insights on the mitochondrial genetic variability of *Lightiella magdalenina* (Crustacea), the sole Mediterranean cephalocarid species

**DOI:** 10.1186/2241-5793-21-5

**Published:** 2014-05-13

**Authors:** Daria Sanna, Alberto Addis, Fabio Scarpa, Francesca Fabiano, Marcella Carcupino, Paolo Francalacci

**Affiliations:** Dipartimento di Scienze della Natura e del Territorio - Sezione di Zoologia, Archeozoologia e Genetica - Università di Sassari, Via Francesco Muroni 25, 07100 Sassari, Italy

**Keywords:** *Lightiella magdalenina*, Mitochondrial genes, Sequencing, Cephalocarida, Mediterranean Sea

## Abstract

**Background:**

Here we report the first insight into the mitochondrial (Cytochrome *c* Oxidase subunit I - COI and Cytochrome b - Cyt b) genetic variation of the only Mediterranean cephalocarid *Lightiella magdalenina.*

**Findings:**

COI sequences provide a scenario of low intraspecific variability, while significant genetic divergence occurs between *L. magdalenina* and *L. incisa*. Interestingly, Cyt b sequences reveal a higher degree of intraspecific variability, with no shared haplotypes between the sites considered.

**Conclusions:**

In the future, COI and Cyt b molecular markers could be used as valuable tools to shed new light into the extant species within the genus *Lightiella* thus providing molecular support to the taxonomical identifications carried out on a morphological basis.

## Findings

### Background

Cephalocarids are a class of small benthic crustaceans distributed from the intertidal zone to a depth of approximately 1550 m [1]. They have been recently considered to be phylogenetically related to the class Remipedia (Crustacea), using both nuclear [2] and mitochondrial [3] markers. Currently, only one order (Brachypoda) has been described with one family (Hutchinsoniellidae) containing twelve species belonging to five genera (*Hutchinsoniella, Lightiella, Sandersiella, Chiltoniella, Hampsonellus*) [4].

The genus *Lightiella* harbours the greatest number of species (five) and the largest distribution (for a review see [4]), with *L. serendipita* Jones, 1961 reported from San Francisco Bay, California, *L. incisa* Gooding, 1963 from the Gulf of Mexico and Caribbean Sea, *L. monniotae* Cals & Delamare Deboutteville, 1970 from New Caledonia, *L. floridana* McLaughlin, 1976 from the western coast of Florida, and *L. magdalenina* Carcupino, Floris, Addis, Curini-Galletti, Castelli, 2006 from Sardinia. However, each species of this genus presents a very restricted distribution, with the exception of *L. incisa,* which is reported to populate the entire Caribbean Sea and the Gulf of Mexico.

The five species belonging to the genus *Lightiella* show limited morphological differentiation [4, 5]. Given the limited number of specimens used for taxonomical classification, their detailed external morphology is often incomplete e.g. [6]. In light of these considerations, Olesen *et al.*[4] advise that the genus needs an urgent overhaul. In particular, these authors suggest that the species richness within the whole Cephalocarida class may have been overestimated. A molecular taxonomic approach could address this problem although molecular data are so far available for only three cephalocarid species: *Hutchinsoniella macracantha*, *L. magdalenina,* and *L. incisa*. Mitochondrial and nuclear genes sequences (see [7–14] for details) are available for *H. macracantha*. Sequences of mitochondrial genes are known for *L. magdalenina* (see [15] for details), while mitochondrial and nuclear gene sequences are known for *L. incisa* (see [3] for details).

The species *Lightiella magdalenina* is the only cephalocarid reported in the Mediterranean and is only found so far from its type locality in La Maddalena Archipelago (Sardinia, Italy) (see [5] for details). Cladistic analysis based on external morphology [5] suggest that *L. magdalenina* is more closely related to the Caribbean species *L. incisa* and *L. floridana* than to the Pacific Ocean species *L. serendipita* (California, Pacific Ocean).

In this study, we provide the first preliminary insight into the genetic variation of the isolated Mediterranean cephalocarid *L. magdalenina* using sequences of the mitochondrial DNA Cytochrome *c* Oxidase subunit I (COI) and Cytochrome b (Cyt b) coding genes from the two close Mediterranean sites where this species is found.

We also provide new specific primers for the COI gene. Results obtained were compared with data from other species of cephalocarids and remipeds.

## Methods

**Figure 1:**
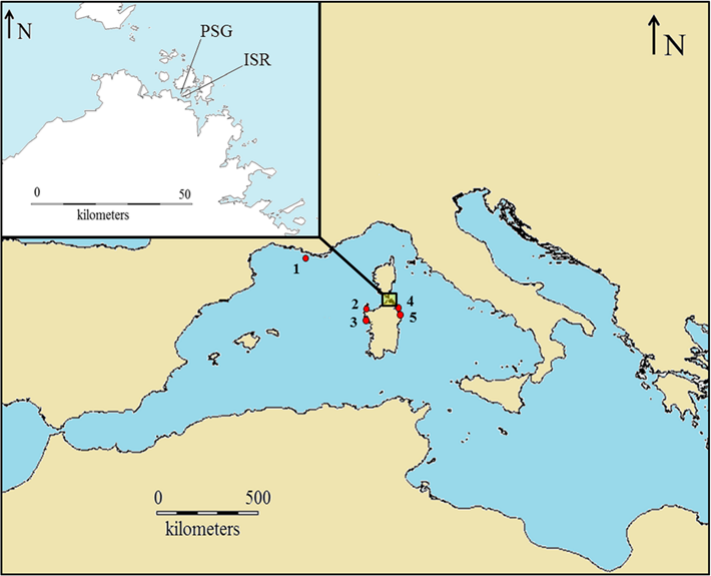
**Map of the sampling sites.** Numbers indicate western Mediterranean zones where no individuals of *Lightiella magdalenina* were found during sampling campaigns. 1: Marseille (3 sites); 2: Stintino (1 site); 3: Alghero (2 sites); 4: Golfo Aranci (1 site); 5: Cala Finanza - Loiri Porto San Paolo (1 site). The boxed area in the map is depicted in further detail in the outset. PSG: Punta San Giorgio (41° 11' 23.9" N; 09° 23' 54.7" E); ISR: Isolotto Roma (41° 11' 43.1" N; 09° 23' 42.3" E).

Eighteen specimens (10 adults and 8 larvae) of *Lightiella magdalenina* were collected at a depth of 15–25 m from muddy sand of the southern shore of La Maddalena Archipelago (Sardinia, Italy) (Figure 1). Sampling was carried out, using SCUBA, during the 2007–2011 spring/summer seasons. We analysed specimens from the type locality (Punta San Giorgio) and from an adjacent site (Isolotto Roma) (approximately 600 m farther), both characterised by bottoms with *Posidonia oceanica* and *Caulerpa racemosa* (see Figure 1 for details). No individuals of *L. magdalenina* were found when sampling campaigns were carried out in other eight sites from five western Mediterranean localities in northern Sardinia and southern France (see Figure 1 for details).

DNA was extracted from whole specimens by means of the QIAGEN^®^ DNeasy Tissue kit (Hilden, Germany). DNA concentration was estimated through fluorometric measurements (average value per sample: 10 ng/μL).

Since COI universal primers for marine invertebrates [16] did not result in good quality sequences, the COI region was amplified using specific primers designed in the present study (L: 5′-gttactctggggggattcgga-3′ and H: 5′-ggccaaaatagaagaaaccccagcta-3′). Universal primers reported by Boore and Brown [17] (L: 5′-ggwtaygtwytwccwtgrggwcarat-3′ and H: 5′-gcrtawgcraawarraartaycaytcwgg-3′) were used for the Cyt b gene. Attempts to design new specific primers for this region, without critically affecting the quality of the amplified fragment, failed. Each 25 μL PCR mixture contained approximately 50 ng of total genomic DNA, 0.4 μM of each primer, and 2.5 U of EuroTaq DNA Polymerase (Euroclone^®^, Italy) in a reaction mix prepared according to the manufacturer’s instructions. The MgCl_2_ concentration was set at 2.5 mM.

PCR amplification was performed with an initial denaturation step of 2 min at 94°C, followed by 35 cycles of 1 min at 94°C, 1 min and 30 sec at 49°C, and 1 min and 30 sec at 72°C. The final elongation step was for 5 min at 72°C with a final cooling at 4°C. In all experiments, negative controls and replicates were included. Electrophoretic runs were carried out at 4 V/cm for 20 min in 2% agarose gels prepared using 0.5 × TBE buffer. The gels were then stained with ethidium bromide (10 mg/mL).

PCR products were purified with ExoSAP-IT (USB Corporation^®^, USA) and sequenced using an external sequencing core service (Macrogen Inc.^®^, Europe). Sequences were aligned using Clustal W [18], implemented in the BioEdit 7.1.3.0 software package [19], and deposited in GenBank [COI:JX013538-JX013555; Cyt b:JX013556-JX013565].

In order to characterise genetic variation among individuals in both populations, the number of polymorphic sites (*S*), of haplotypes (*H*), the estimates of haplotype diversity (*h*), and nucleotide diversity (*π*), were computed using the software DnaSP 5.10 [20].

Genetic relationships among haplotypes were investigated by constructing a median-joining network using the software Network 4.6 [http://www.fluxus-engineering.com/]. In the absence of random recurrent polymorphisms across the whole dataset, an identical weight (10) was given to all mutations to preserve the original genetic information. We ran the Maximum Parsimony (MP) calculation post-processing option to delete all superfluous median vectors and links that were not contained in the shortest trees of the network. For the COI gene, the analysis was performed by adding also the sequences of two other cephalocarids, *Lightiella incisa* [GenBank:GQ328968] and *Hutchinsoniella macracantha* [GenBank:AF370852]. In addition, two sequences, representative of the two families of the class Remipedia, *Pleomothra apletocheles* (Godzilliidae) [GenBank:GU067682] and *Speleonectes tulumensis* (Speleonectidae) [GenBank:NC_005938], were included in the analysis as outgroups. For the Cyt b gene, the analysis was carried out including the cephalocarid *Hutchinsoniella macracantha* [GenBank:AY456189] plus the remipede *Speleonectes tulumensis* [GenBank:NC_005938] as outgroup.

## Results

### COI analysis

Eighteen individuals were sequenced and a 216 base pair (bp) alignment was obtained. A previously published sequence [GenBank:EU530536] [15] belonging to an individual from Punta San Giorgio (see Table 1 for details) was added to the dataset. All 19 specimens shared the same haplotype, except one specimen from Isolotto Roma [GenBank:JX013545], bearing a single non-synonymous transversion (G to C). Indices of genetic variation are reported in Table 1.

**Table 1:** **Estimates of genetic diversity for COI and Cyt b gene fragments**

Sample	N	bp	***S***	***H***	***h***	***π***
**COI**						
Punta San Giorgio	5	216	0	1	0.000	0.00000
Isolotto Roma	14	216	1	2	0.143	0.00066
Total	19	216	1	2	0.105	0.00049
**Cyt b**						
Punta San Giorgio	3	348	8	2	0.667	0.01533
Isolotto Roma	7	348	2	2	0.476	0.00274
Total	10	348	10	4	0.733	0.00888

N: sample sizes; bp: base pairs; *S*: number of polymorphic sites; *H*: number of haplotypes; *h*: haplotype diversity; *π*: nucleotide diversity.

**Figure 2:**
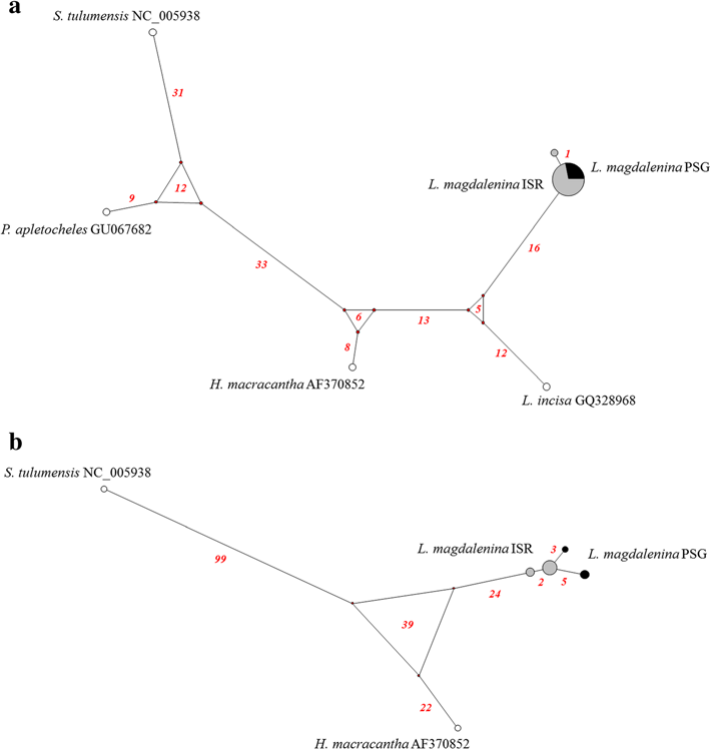
**Median-joining network showing relationships among** ***Lightiella magdalenina*** **haplotypes and other cephalocarids and remipedes.** Median-joining network based on COI **(a)** and Cyt b **(b)** datasets. The number of point mutations is reported along the lines connecting haplotypes. Rings on the nodes show median vectors, representing hypothetic connecting sequences, calculated with the maximum parsimony method. Pie sizes are proportional to the absolute frequency of each haplotype/sequence. Due to graphical representation issues the length of edges connecting *L. magdalenina* haplotypes is not exactly proportional to the number of mutating positions between haplotypes. The sample codes are reported in Figure 1. For species abbreviations, refer to the main text.

Results obtained by the median-joining network analysis (Figure 2a) provided support for strong genetic differentiation between *L. magdalenina* and *L. incisa.* Sixteen (for *L. magdalenina*) and 12 (for *L. incisa*) point mutations represent the apomorphic polymorphisms of the two species. Twenty-seven point mutations separated *H. macracantha* from the group including *L. magdalenina* and *L. incisa.*

### Cyt b analysis

As a possible consequence of the limited homology between the universal primers [17] and the annealing region in our specimens, scorable Cyt b sequences were obtained for only ten individuals (three from Punta San Giorgio and seven from Isolotto Roma). The already published Cyt b sequence [GenBank:EU530537] was not included in the dataset since a BLAST search revealed highest similarity with *Saprolegnia ferax* (a fungus belonging to the family Saprolegniaceae) (81% identity, coverage 87%) and indicated a possible fungal contamination of this sample. A 348 bp long alignment was obtained. In total, four haplotypes were detected. Values of genetic diversity were higher than those obtained for the COI gene (Table 1). No haplotypes were shared between Punta San Giorgio and Isolotto Roma samples. All of the ten polymorphic sites retrieved were biallelic with seven synonymous substitutions. Eight transversions and two transitions occurred.

The median-joining network analysis (Figure 2b) revealed a well-defined divergence, supported by 85 to 125 point mutations (this pattern contains a cycle with three median vectors separated from each other by 39 mutational steps) between *L. magdalenina* and *H. macracantha.* Within the *L. magdalenina* group, the most common haplotype was exclusive of individuals from Isolotto Roma, while two haplotypes belonging to Punta San Giorgio and one from Isolotto Roma diverged by two to five point mutations from the root sequence.

## Discussion

Five decades after the first description of Cephalocarida [21] our knowledge of this taxon is still limited, especially for species other than *Hutchinsoniella macracantha*. The present work evidences that if COI universal primers [16] fail, even a 216 bp long internal portion (amplified by the new primers provided here) of the widely used 710 bp long Folmer [16] region can be used for the taxonomic identification of the species belonging to the genus *Lightiella*. Consistently, previous studies [22, 23] showed that partial fragments of the Folmer region of about or less than two hundred bp may correctly assess phylogenetic/phylogeographic traits of the species. In such a context, our preliminary analyses provide molecular support to the morphology-based distinction reported between *L. magdalenina* and *L. incisa*[5].

Sequencing of the Cyt b gene, performed on *L. magdalenina* specimens, showed higher values of intraspecific genetic variability than the COI gene. Indeed, the absence of shared Cyt b haplotypes between *L. magdalenina* specimens from the two neighbouring sites sampled in this study is consistent with the possible occurrence of two distinct populations. In spite of the close geographical proximity of the two sites (only 600 m apart), the isolation can be explained by restricted population size [24] together with a low dispersion capacity of the species [1]. In fact, *L. magdalenina*, lacks a high-dispersal phase, given that both larval stages and adults show a strictly benthic lifestyle [1, 25]. However, it should be taken into consideration that this pattern could be affected by the small number of samples from Punta San Giorgio (three individuals), thus preventing supported inferences.

In conclusion, this study reports the first data on the genetic variation of a cephalocarid species, providing a preliminary survey on the mitochondrial variability of the only Mediterranean species, *L. magdalenina.*

The high level of interspecific genetic divergence reported between *L. magdalenina* and *L. incisa,* using the COI gene, offers molecular support to previous morphological analysis [5], allowing to consider this marker as a valuable tool for molecular taxonomic assays within the genus *Lightiella.* On the other hand, if confirmed by further analysis on a larger number of samples, the estimates of intraspecific variation based on the Cyt b gene for *L. magdalenina* suggest the possible use of this marker for assessing the phylogeographic patterns of the species.

These results highlight the importance of performing joint (COI and Cyt b) molecular analyses in cephalocarids for further taxonomic revision of the genus *Lightiella* and population genetic surveys.
